# Portable Diffuse Reflectance Spectroscopy of Potato Leaves for Pre-Symptomatic Detection of Late Blight Disease

**DOI:** 10.1177/00037028231165342

**Published:** 2023-03-17

**Authors:** Chen Zhou, Victor G. Bucklew, Perry S. Edwards, Chenji Zhang, Jinkai Yang, Philip J. Ryan, David P. Hughes, Xinshun Qu, Zhiwen Liu

**Affiliations:** 1 Department of Electrical Engineering, 8082 The Pennsylvania State University, University Park, PA, USA; 2 Atoptix, Inc., State College, PA, USA; 3 Department of Entomology, 8082 The Pennsylvania State University, University Park, PA, USA; 4 Department of Plant Pathology and Environmental Microbiology, The Pennsylvania State University, University Park, PA, USA

**Keywords:** Diffuse reflectance spectroscopy, DRS, miniature spectrophotometer, machine learning, pre-symptomatic plant disease detection, potato late blight

## Abstract

We report on the use of leaf diffuse reflectance spectroscopy for plant disease detection. A smartphone-operated, compact diffused reflectance spectrophotometer is used for field collection of leaf diffuse reflectance spectra to enable pre-symptomatic detection of the progression of potato late blight disease post inoculation with oomycete pathogen *Phytophthora infestans*. Neural-network-based analysis predicts infection with >96% accuracy, only 24 h after inoculation with the pathogen, and nine days before visual late blight symptoms appear. Our study demonstrates the potential of using portable optical spectroscopy in tandem with machine learning analysis for early diagnosis of plant diseases.

## Graphical abstract

**Graphical Abstract:**
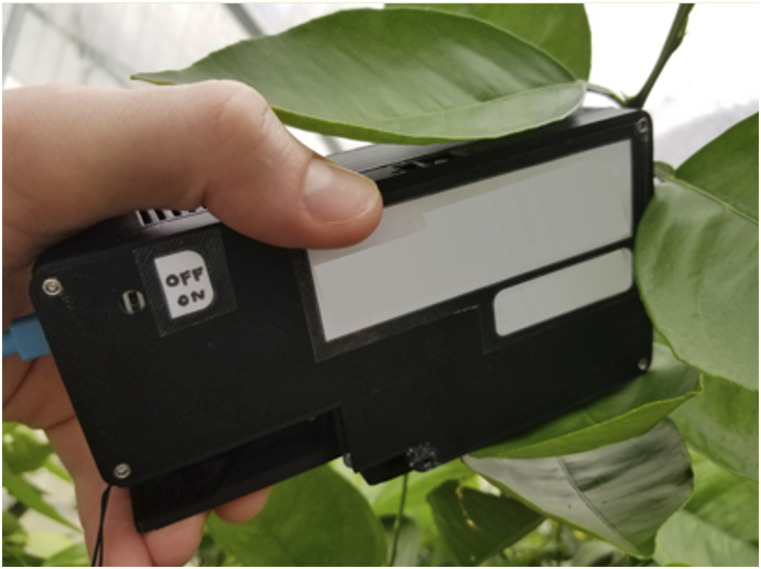
This is a visual representation of the abstract.

## Introduction

Diffuse reflectance spectroscopy (DRS)^1^ is a non-invasive diagnosis technique that captures optical spectra indicative of the scattering and absorption characteristics within a sample. By spatially separating light illumination and signal detection, DRS only collects light that has undergone scattering and absorption inside a specimen and can thus interrogate the interior as opposed to the surface of the specimen. With this unique capability, DRS has found important applications in the biomedical field, such as probing human adenomatous colon polyps,^2^ and the diagnosis of breast cancer^3^ and nonmelanoma skin cancer.^4^ DRS has also been applied to plant health applications, such as determination of the chlorophyll content^5^ in plant leaves and monitoring of peach quality deterioration caused by fungi infection.^6^

The importance of developing portable optical spectroscopy for plant disease detection cannot be overstated. Plant diseases are estimated to reduce worldwide crop production by 16%,^7^ posing a serious threat to global food security. Plant diseases are often diagnosed by analysis of leaves, since a broad range of plant diseases including fungal, bacterial, and viral diseases result in characteristic changes in leaves.^8^ For example, visual inspection or scouting^9^ of leaves is arguably the most commonly practiced method; however, it is prone to human error and can only detect diseased leaves with visible symptoms. Laboratory based molecular detection techniques,^10,11^ such as polymerase chain reaction testing, are powerful but are too costly to be deployed at large scales and challenging to be deployed in field. Optical spectroscopy has been demonstrated as an effective means for detecting nutrient, water, disease stresses^12–16^ in plants, as well as quantifying foliar structural, phytochemical, and morphological properties^17–21^ in plants. Furthermore, optical spectroscopy was applied for in-field plant disease detection. For example, reflectance spectroscopy was reported to enable pre-symptomatic detection and discrimination of *Phytophthora infestans* caused underlying biochemical and physiological processes in potato, from measured reflectance spectra on potato plant leaves at early infection stage.^22,23^ However, many existing methods involve bulky and high-cost instruments.

**Figure 1.**
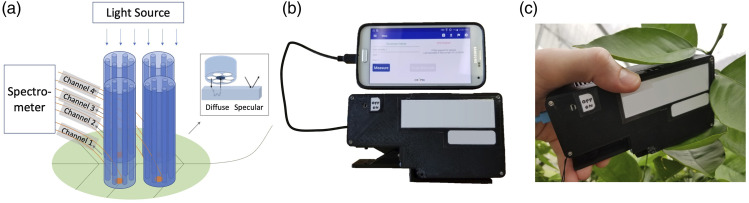
Smartphone-based spectrophotometer. (a) Schematic depicting the diffuse reflectance spectroscopy modality implemented using four fiber probes (channels), each of which consists of six surrounding light delivery fibers and a central fiber for collecting the diffuse reflectance signal. In comparison, surface-reflection-based measurements do not penetrate into the insides of a sample (inset). (b) Smartphone-based spectrophotometer prototype. The handheld spectrophotometer is connected to a smartphone with a micro-USB cable. A smartphone app can initiate a measurement and store the data. (c) Photo showing the sensor in use, performing in-field non-invasive leaf DRS measurement.

Current plant DRS data acquisition is usually conducted in laboratory settings where bulky instrumentation and skilled operators are often required, making practical, large scale field deployment with low lead-time actionable results very challenging. The application of low-cost, lightweight (<454g or <1 lb.), compact, and field-deployable DRS sensors to in situ plant health assessment, in particular early detection of plant diseases, remains not extensively explored. Here, we report on the development of a handheld DRS sensor and an efficacy study of in-field leaf DRS measurements for detecting late blight disease caused by oomycete pathogen *Phytophthora infestans* in potatoes before the onset of visual symptoms. With the capability to probe the interior of a leaf (Fig. 1a, inset), the diffuse reflectance modality could provide an added sensitivity to detecting plant diseases during a pre-visual stage of infection in which modification to plant structures by the disease has already begun in the leaf, but where it has not yet reached a severity where it can be seen on the outer surface of the leaf. The late blight potato disease is selected in this study due to both its economic impact and the importance of early detection: it has been conservatively estimated to be responsible for US$6.4 billion in annual losses.^24^ The handheld DRS spectrophotometer sensor was used in conjunction with a smartphone to enable scalable collection of DRS data in the field and to provide a convenient means for data labeling (including time stamping and global positioning system coordinate recording) and storage.

Analytical modeling^2^ or Monte Carlo-based^25^ inversion is typically used to analyze biomedical DRS data. However, extending these inversion methods to leaf DRS analysis may require considerations of significant property differences between leaves and biomedical tissues. To mitigate the challenges associated with physical model-based approaches, a data driven approach^26,27,28^ is used to infer the health status (diseased or healthy) from measured DRS data. We discuss the efficacy of the DRS inference when binary and multiclass neural network classification algorithms are used, demonstrating that DRS can detect disease well before visual symptoms appear.

## Materials and Methods

### Diffuse Reflectance Spectrophotometer

A handheld, smartphone-operated, diffuse reflectance spectrophotometer sensor was designed and developed for pre-symptomatic detection and quantification of crop diseases. Figure 1a shows a schematic of the spectrophotometer system. The DRS spectrophotometer has four measurement channels for sampling four positions on a leaf (two on each side of the midrib). In each measurement channel, light from a built-in micro-halogen light source is coupled into an array of six optical fibers, which are arranged in a circular pattern around a single receiving fiber (Fig. 1a, inset). The receiving fiber, located in the center, is internally coupled to a miniature spectrometer. The spectrometer has a wavelength measurement range from 400 to 1000 nm and a resolution of 5–7 nm and contains a mobile camera module with a complementary metal oxide semiconductor (CMOS) image sensor. Leveraging the two-dimensional CMOS sensor, the four receiving fibers from all channels are routed to be positioned along the slit of the spectrometer so that the DRS spectra from all four locations can be captured simultaneously. In addition, the spectrum of the micro-halogen light source is also monitored by using another fiber to pick off some of its light; this fiber is also routed to the spectrometer and positioned together with the four receiving fibers. During each measurement, five spectra are captured simultaneously: one being the micro-halogen source reference spectrum and the others being the four DRS spectra, yielding four self-referenced DRS measurements per leaf scan. The core size of the fiber is 400 μm, covering an area of ∼0.126 mm^2^ on the plant leaf by each measurement channel. The spectrophotometer system also has micro-electronic control circuitry to operate both the spectrometer and the lamp source. Contact with the surface of a leaf sample is made with a leaf clip attachment (Fig. 1c) that holds the leaf tight against the four measurement channels. After contact is made, light from the illumination fibers penetrates into the leaf and is scattered and absorbed by inner leaf structures. Some of the light is eventually collected into the receiving fibers, carrying spectroscopic information indicative of the light-leaf interaction (i.e., absorption and scattering), and is measured by the spectrometer. Since the measured DRS spectra are dependent on the inner leaf structures, which are expected to change due to disease infection and progression, a DRS spectrophotometer sensor can provide correlative spectroscopic information to assess plant health.

The spectrophotometer was tethered to a Samsung Galaxy S5 smartphone using a micro-USB cable that communicates with the spectrophotometer using USB on-the-go (OTG) protocols. An Android application (app) was configured to initiate a spectral capture sequence and to receive and store captured spectral data on the smartphone for later download and processing on a personal computer. The Android app can turn the built-in micro-halogen lamp source on or off as part of the capture sequence and configure the lamp illumination time and the detector integration time.

### Field Measurements

Twenty-six potato cultivars and advanced breeding lines were evaluated for late blight susceptibility at the Pennsylvania State University Russell E. Larson Agricultural Research Center in Pennsylvania Furnace, Pennsylvania, USA. The soil type was a Hagerstown silty clay loam. Potatoes were planted on 9 June 2020. The experimental design was a randomized complete block with three replicates. The plots were 1.21 m long with five seed pieces planted in each plot and 1.52 m breaks between plots within a row. Each treatment row had an adjacent row of the susceptible cultivar. Atlantic as a spreader row. On 23 Aug 2020, spreader rows were spray-inoculated with a mixture of four isolates of *Phytophthora infestans* clonal lineage US-23, at a concentration of 2.1 × 10^5^ sporangia/mL, to promote a uniform spread of the pathogen to all treatment plots. Overhead sprinklers were used for approximately one hour daily when the weather was dry and hot to increase humidity in the plant canopy after infection. We irrigated using overhead sprinklers before and after inoculation. The irrigation water was evenly distributed on all plants in the field including healthy and unhealthy plants. Late blight disease pressure was high, and the most susceptible plots reached 100% disease severity by the end of the study. Standard crop management practices were followed throughout the growing season, with the exception that no fungicides were sprayed for late blight.

*Phytophthora infestans* can rapidly destroy potato plants under favorable environmental conditions.^22^ During early infection phase, the pathogen spreads throughout the plant tissue while feeding on living cells without producing visual symptoms. Visual necrotic lesion symptoms typically occur several days after infection. Although there are no visible symptoms during early infection phase, late blight infection changes potato foliar biochemical, physiological, and structural properties. Leaf water content is changed, and cellulose is compromised by late blight infection. Infected leaves have greater phenolics and nitrogen concentrations and lower sugar and starch concentrations than healthy leaves. These structural and chemical changes contribute to the modification of optical properties of the leaf. Within 5–7 days, depending on the temperature (optimum at 15–21 °C), visible lesions can develop from these infections. New sporangia are continually produced as long as humidity is high, and the lesions are viable. Sporangia dislodged from leaf or stem lesions due to changes in environmental conditions (e.g., humidity, wind, irrigation, water, or rainfall) can move downward from the crop canopy, causing new infections on either the stems and leaves of the same plant, or a different plant. Sporangia can also fall onto the soil, increasing the risk of tuber infections, or land directly on exposed tubers, resulting in infection.^29–31^

Diffuse reflectance leaf measurements of potato cultivar Atlantic were longitudinally performed with the DRS spectrophotometer. The integration time on the spectrophotometer was controlled with the Android app and set to a constant value of 2 s for the duration of the experiment. Each measurement sequence (the time taken to clip the sensor onto the leaf, initiate a measurement with the Android app, and complete the measurement) took an average of a few seconds. For the duration of the experiment, approximately 600 spectral measurements were collected each day evenly distributed amongst six plants. The cultivar of the six plants is Atlantic. Each time, about 25 leaves were selected from each plants and spectra were non-invasively collected from both the top and bottom parts of the plant to avoid bias. Four measurements on the adaxial side of the mid-vein were captured simultaneously for each leaf using the sensor, with two measurements closer to the leaf tip, and two closer to the leaf base. Spectral readings were taken with the light source penetrating through the adaxial surface of the leaf, and diffuse reflectance measurements were collected similarly from the same side of the leaf. The micro halogen illumination lamp was replaced after about every 1000 measurements in order to minimize the impact of lamp degradation on signal to background levels and that they remained above a threshold level.

**Figure 2.**
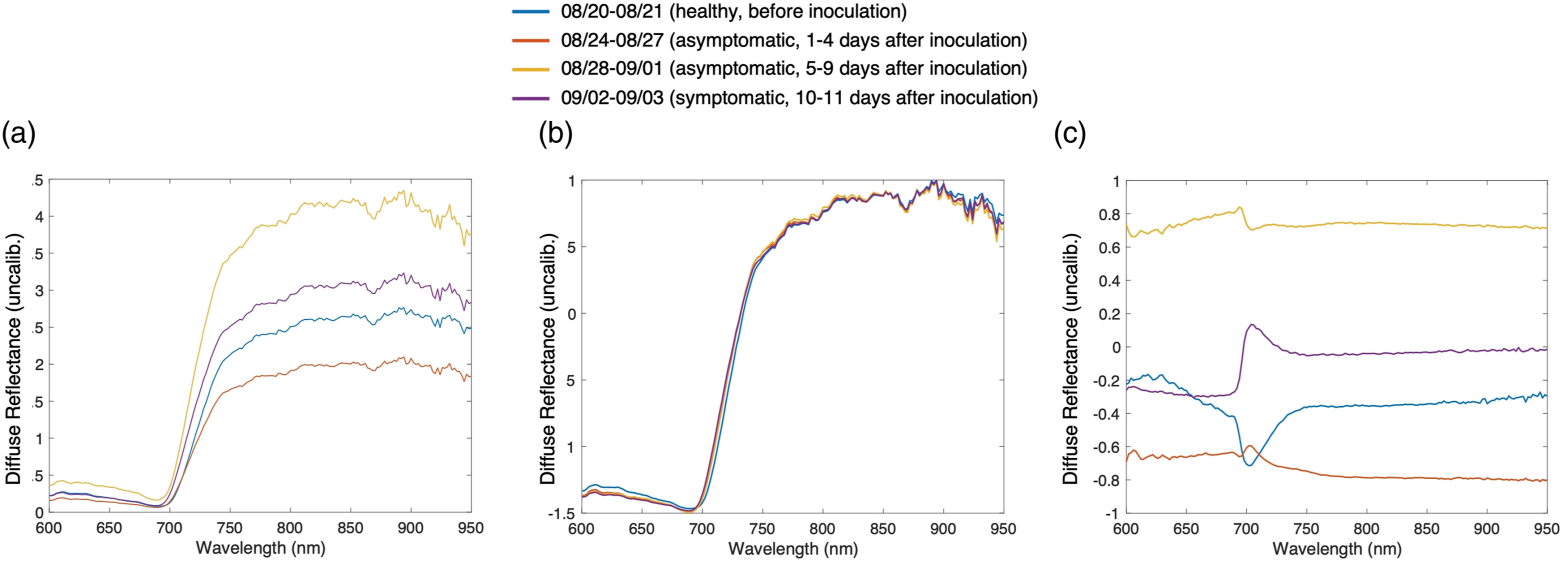
Averaged diffuse reflectance spectra over time periods within the study, showing the correlation between the spectral signature and the disease progression within the plant. Each spectral trace reflects the average of all measurements collected within the time period. Inoculation: Plants were sprinkler inoculated with *Phytophthora infestans* in the evening of 23 August 2020. Visible Symptoms: The first visible symptoms appeared on 2 September 2020. (a) Before normalization, (b) after normalization of each measurement for all wavelengths, and (c) after normalization of each wavelength for all measurements. Normalization parameters in (c) are calculated from four plants that were used for building the machine learning model.

Table I summarizes the number of spectra after pre-processing the data for each of the 13 days that leaf measurements were collected. Figure 2 shows several averaged diffuse reflectance spectra for selected dates. Compared to the near infrared wavelengths, the visible portion (∼600–700 nm) of the signal is weaker primarily due to significant absorption by chlorophyll.^32,33^ However, the spectrophotometer also captures the near infrared part of the spectrum, invisible to human eyes, providing broader spectral information for disease quantification and diagnosis. In Fig. 2, the blue curve is the averaged spectrum of the healthy (before inoculation), while the other three correspond to early (the first four days after inoculation), middle (days 5–9), and late stage (days 10 and after) infection, respectively. Visible symptoms appeared during the late stage (corresponding to the purple curve). These averaged spectra demonstrate clear differences between the “healthy” state (before the inoculation) and the “infected” state (after the inoculation). The average diffuse reflectance signal intensity at the early stage of the infection was in general lower compared to that of the healthy leaves. As the infection progressed, the average intensity became higher but dropped again toward the late stage. Leaves from both mid and late stages had higher average diffuse reflectance intensity compared to the healthy and early infection stages.

**Table I.** Summary of number of spectra captured in each day of measurement (after outlier removal).

Healthy class count	20-Aug	21-Aug								Total
	677	594								1271
Asymptomatic class count	24-Aug	25-Aug	26-Aug	27-Aug	28-Aug	29-Aug	30-Aug	31-Aug	01-Sept	Total
	436	435	441	463	496	482	510	479	488	4230
Symptomatic class count	02-Sep	03-Sep								Total
	480	468								948

### Data Analysis

The data analysis includes removal of outliers, data preprocessing, implementing, and comparing machine learning algorithms, and building binary or multiclass classification models. First the outliers are removed. We find that outliers are generally caused by either improper contact between the sensor and the leaf, or from sensor timing malfunctions resulting from sensor-app miscommunication. The outliers are detected based on a median absolute deviation (MAD) algorithm where outliers are defined as values more than three-scaled MAD from the median.^34^ The spectroscopic data is then normalized with the *z*-score method.^35^Figure 2a shows the average DRS spectra corresponding to different time periods before normalization. Normalization along different dimensions (measurement, wavelength) is performed. Figure 2b shows the intensity after normalization of each individual measurement to be centered and scaled to have a mean of 0 and a standard deviation of 1. Figure 2c shows the intensity after normalization at each wavelength for all measurements. The latter shows better separation among different stages. Therefore, the method of normalization across measurements is selected and utilized for the following analysis. Normalization parameters are calculated from plants that are used to build the model. Spectra from new plants that are tested with the model are directly normalized using the parameters from the training plants.

Different machine learning algorithms (i.e., decision tree,^36^ linear discriminant,^37^ support vector machine (SVM),^38^ logistic regression,^39^*k*-nearest neighbors (KNN),^40^ and neural network^41^) are implemented on the dataset, and accuracy results are compared, to predict the health state based on the pre-processed spectra. First, we begin by dividing the combined spectroscopic data for all days into two categories. The data collected on and after the 24 August 2020 (24 h after inoculation) is classified as unhealthy, while the data on the days before the 24 August, which includes all of the days before inoculation, is classified as healthy. We use spectral data from leaves from four plants to build the machine learning model and test it with spectral data of two separate plants as the testing set to validate the robustness of the trained model. In building the neural network model, we randomly select 90% of the data of the 4 plants from each category as the training set, 5% as the validation set, and 5% as the testing set. The validation set is used to validate the training in every iteration to prevent over-fitting through the early stop validation algorithm, and the testing set is to preliminarily verify the predictive power of the network. After the network is trained and its parameters are fixed, we use the leaf spectral data from the two other separate plants, which are not seen during the model-building phase, to test the robustness of our learning model and characterize the model accuracy.

## Results and Discussion

Comparison of different machine learning algorithms are shown in Table II. Overall, for all six algorithms, the accuracy on the separate two plants that are unseen by the model is at least >87%. Among them, neural network has the best performance in terms of the accuracy. Test accuracy on four trained plants is 98.6% and test accuracy on two new plants is 96.4%. Therefore, neural network is selected to build the model for binary and multiclass classification in the following analysis.

**Table II.** Comparison of different machine learning algorithms.

Algorithm	Accuracy^a^	Accuracy^b^
Decision tree	88.1%	87.3%
Linear discriminant	96.7%	93.9%
Support vector machine (SVM)	95.2%	90.1%
Logistic regression	96.2%	94.2%
K-nearest neighbors (KNN)	89.0%	88.7%
Neural network	98.6%	96.4%

^a^Measurements of four plants for building the models are divided into training (90%), validation (5%), and test (5%). Accuracy showed here refers to the test group.

^b^Trained model is applied to two other new plants, and accuracies are reported here.

**Figure 3.**
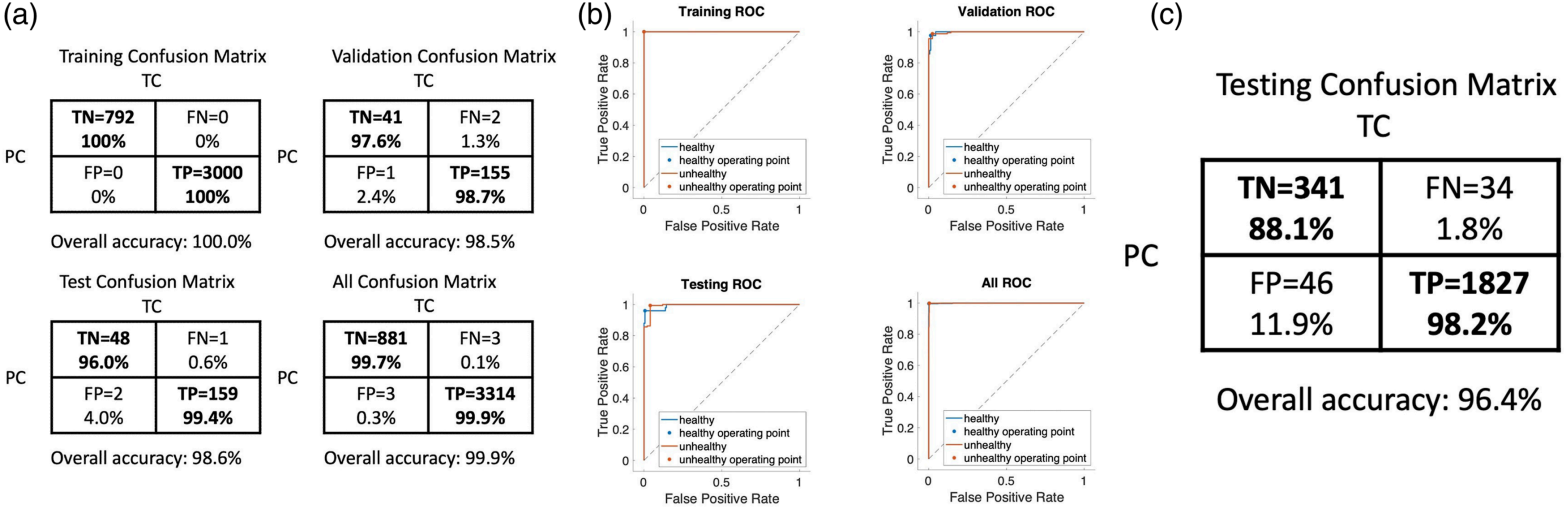
Binary classification results. (a) Confusion matrix for the training, validation, testing, and overall data, respectively. TC: true condition, PC: predicted condition, TN: true negative; samples that are healthy and classified as healthy, TP: true positive, samples that are unhealthy and classified as unhealthy, FN: false negative, samples that are unhealthy but classified as healthy, FP: false positive, samples that are healthy but classified as unhealthy. (b) ROC curves for the training, validation, testing, and overall data, respectively. (c) Confusion matrix for the testing with two separate potato plants using the trained model.

Results of the neural network machine learning algorithm are presented in Fig 3. Positive is defined as symptomatic and negative is healthy. The boxes with true positive (TP), true negative (TN) in the confusion matrices represent correct predictions, and the false positive (FP) and false negative (FN) represent incorrect ones. The analysis yields an overall accuracy of 96.4%, true positive rate of 98.2% and a true negative rate of 88.1% on testing sets of separate plants. The receiver operating characteristic (ROC) curves for the training, validation, testing, and overall data are also plotted in Fig. 3. The areas under the curves are nearly unity, indicating that both high sensitivity and high specificity can be achieved.

**Figure 4.**
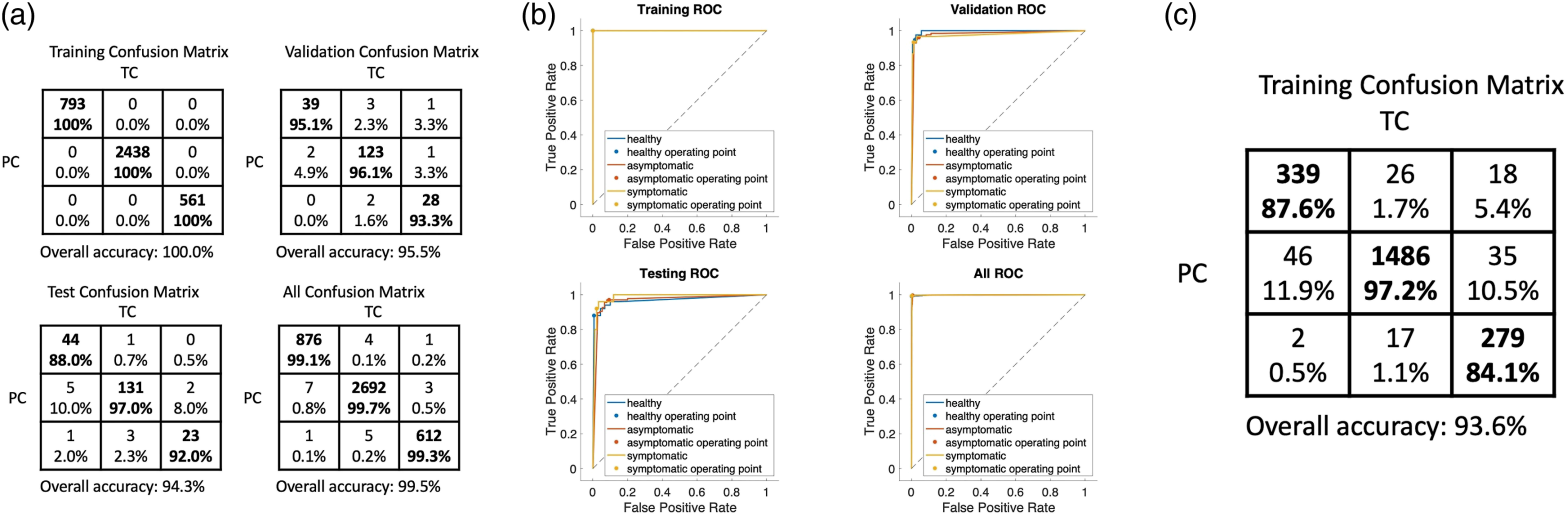
Multiclass classification results. The spectral data are classified into three classes (healthy, asymptomatic, symptomatic). (a) Confusion matrix in which TC: true condition (healthy, asymptomatic, symptomatic from left to right columns, respectively), PC: predicted condition (healthy, asymptomatic, symptomatic from top to bottom rows, respectively). (b) ROC curves are presented for the training, validation, testing, and overall data, respectively. (c) Confusion matrix for the testing with two separate potato plants using the trained model.

Next, we considered whether we could translate the performance of the two-class to a multiclass classification that would allow us to quantify not only if a plant is healthy or diseased, but also at what stage of infection the plant was in. Such information can be critical for growers for designing appropriate interventions. We split the spectral data into three classes corresponding to the healthy (before inoculation), asymptomatic (1–8 days after inoculation when visual symptoms had not yet appeared), and symptomatic health states of the plants. Same as before, we use four individual plants for building the multiclass neural network, and we test the robustness of the model with two other independent plants and characterize the model accuracy. The multiclass classifier has an accuracy of 87.6% for correctly predicting the healthy plant state, 97.2% accuracy for correctly predicting the asymptomatic plant state, and an 84.1% accuracy for correctly predicting the symptomatic plant state (Fig. 4). Although the accuracy for predicting the symptomatic plant state is slightly lower, most of the error is due to predicting the plant state as asymptomatic, rather than the healthy state. The ROC curves for the training, validation, testing, and overall data are plotted in Fig. 4, approaching ideal ROC curves.

For the binary classifications, we further explored the requirement of the number of the potato plants needed to effectively train the machine learning model in order to accurately predict the crop health status. We randomly select *n* (*n* = 1, 2, 3,4,5) potato plant(s) as the training set, and the other separate plant(s) as the test set to characterize the model accuracy. Results of these studies are presented in Table II. Intriguingly, it is possible to train the model with leaves from only one potato plant and still obtain high accuracies in distinguishing between the various classes by testing on the other separate five potato plants, indicating that the model is robust, and the system has a high sensitivity. With more plants in the training set to build the model, the trending of the testing accuracy increases as expected. The high accuracy is demonstrated in both true positivity rate and true negative rate. Note the learning model variance contributes to deviations from the central tendency.^42,43^ In Table III, for the cases of training with one and two plants, the accuracy is within the model variance. Similarly, the accuracy is also within the model variance when four and five plants are used to train.

**Table III.** Comparison of models trained with different numbers of potato plants and testing results.

Data set (%)		Binary classification accuracy (%)		
Training (plants)	Testing (plants)	True positive rate	True negative rate	Overall accuracy
1	5	94.9	77.7	91.6
2	4	97.6	86.1	95.4
3	3	99.1	85.6	96.5
4	2	98.2	88.1	96.4
5	1	99.7	78.3	96.1

For additional confirmation that the device is cueing in on spectral changes resulting from the presence of the pathogen and progression of the disease, we analyzed the data for each day in which spectral measurement was performed and computed the daily positive predictive rate (DPPR) of the disease, which is defined below as

(1) $$DPPR=\frac{TP}{TP+FP}$$

in which TP is the true positive and FP is the false negative.

**Figure 5.**
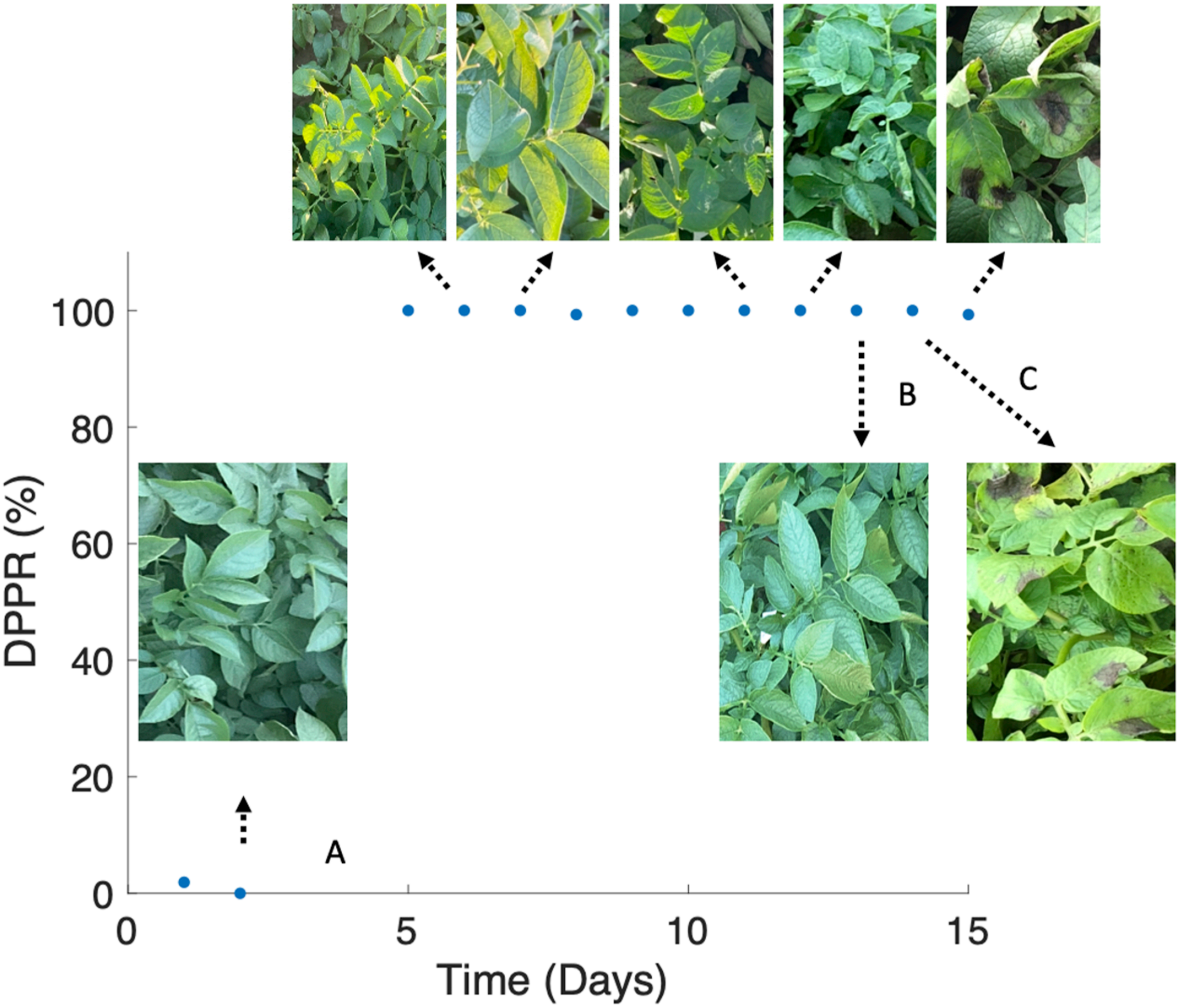
DPPR indicating the sensitivity of the spectrophotometer system to the progression of potato late blight disease. The spectrophotometer is sensitive to late blight just one day after inoculation and predicts the presence of the disease nine days before plants showed any visual symptoms of being infected. A: Day of inoculation; B: last day after inoculation that plants were asymptomatic; C: first day after inoculation that plants displayed visual symptoms of late blight.

As shown in Fig. 5, although symptoms did not visibly appear until the ninth day after the inoculation (2 September 2020) when few necrotic/brown lesioned areas start to appear on some plant leaves, the spectrophotometer could detect spectral changes 24 h after the inoculation with a high daily positive predictive rate of disease. Note that none of the measurements reflect leaf areas with lesion. Only structurally intact sections of the leaf are measured throughout this study. The lesion areas shown in Fig. 5 are only to demonstrate which day in the study the first visual symptoms appeared.

## Conclusion

Early detection and quantification of crop diseases in fields is an important step to develop integrated approaches to control the diseases. This work reports on the efficacy of a handheld smartphone spectrophotometer system, which operates using the diffuse reflectance spectroscopy modality, for high throughput detection and quantification of late blight disease in potato in an asymptomatic stage of the disease development. The high accuracy of the neural network model in distinguishing between healthy and diseased data sets, as well as a multiclass system for more sensitively describing the trend of disease progression, suggests that the diffuse reflectance spectrophotometer platform may be able to provide an important tool for early optimizing interventions for mitigating crop loss from disease pressures. Further, the sensitivity of the platform in detecting spectral changes correlated with disease progression, and then subsequent prediction of these trends in unknown leaf samples with minimal training data, suggests that the platform could potentially be used as a practical tool for pre-symptomatic screening of crop diseases.
